# The epidemic of stroke mortality attributed to high body mass index in mainland China: Current trends and future prediction

**DOI:** 10.3389/fpubh.2022.1021646

**Published:** 2022-10-24

**Authors:** Zhaoqing Du, Wenxuan Zhu, Yuqi Zhao, Shenghang Liu, Hao Chu, Zhonghe Sun, Meng Chu

**Affiliations:** ^1^Department of Hepatobiliary Surgery, Shaanxi Provincial People's Hospital, Xi'an, China; ^2^Health Science Center, Xi'an Jiaotong University, Xi'an, China; ^3^School of Urban Planning and Municipal Engineering, Xi'an Polytechnic University, Xi'an, China; ^4^Nanjing First Hospital, Nanjing Medical University, Nanjing, China; ^5^The First Affiliated Hospital of Xi'an Jiaotong University, Xi'an, China

**Keywords:** obesity, high body mass index, stroke, mortality, Global Burden of Disease Study

## Abstract

**Background:**

High body mass index (BMI) is an important risk factor for stroke. The aim of this study was to assess the long-term trend of high BMI-attributed stroke mortality and make projections through 2030.

**Methods:**

Data were extracted from the Global Burden of Disease Study 2019 and World Population Prospects 2019. An age-period-cohort framework was used in the analysis.

**Results:**

From 1990 to 2019, the age-standardized mortality rate (ASMR) of high BMI-attributed stroke among females decreased by 15.2%, while among males, it increased by 31.1%. All of the age groups studied showed an increasing pattern over the last 30 years in males, and in female, the age groups encompassing participants who were 25–69 years old showed a decreasing pattern. In the same birth cohort, high BMI-attributable stroke mortality rates increased exponentially with age in both sexes. For females, the period rate ratios (RR) showed a downward trend after 2000–2004, and the cohort RR also showed a downward trend after the birth cohort 1930–1934. For males, the period RR showed an upward trend, but this increase was halted in the most recent period, and the cohort RRs showed a monotonic increasing pattern. It was projected that the ASMR of high BMI-attributed stroke would decrease among females and increase among males in the near future and that the proportion of elderly individuals with death due to high BMI-attributed stroke was projected to increase.

**Conclusions:**

Over the last three decades, the high BMI-attributed stroke mortality rate decreased among females and increased among males, and these trends are projected to continue in the future. In addition, the proportion of elderly individuals with high BMI-attributed stroke mortality was projected to increase gradually in both men and women. More health-promoting efforts are needed, especially for elderly individuals and males.

## Introduction

Globally, stroke is the second leading cause of death in the elderly population and the fifth leading cause of death of middle-aged people (1). China accounts for approximately one-third of global stroke deaths (2), as the situation there is even worse. It is estimated that there are over 2 million new cases of stroke annually (3), ~115 per 100,000 (3), which has exceeded the incidence of heart disease to become the leading cause of death and adult disability (1). In 2018, 1.57 million people died of stroke in China, accounting for 22.33% of all deaths (4).

High body mass index (BMI) is an important risk factor for stroke (5). Approximately 16.4% of the global age-standardized mortality rate (ASMR) of stroke is caused by high BMI in 2017 (6). There is a significant J-shaped dose–response relationship between BMI and the incidence of stroke (5); for every 5 kg/m^2^ increase in BMI, the risk of stroke increases by ~10% (5). Over the past four decades, the number of adults in China with obesity (BMI: 28.0 kg/m^2^ or higher) has more than quadrupled, and the number with overweight (BMI: 24.0–27.9 kg/m^2^) has more than doubled (7). According to the most recent national data (2015–19), more than half of Chinese adults have a high BMI (BMI above 24.0 kg/m^2^) (7). To make matters worse, the number of obese people is expected to continue to increase (8). Obesity already has a tremendous impact on the burden of stroke in China.

Previous studies have evaluated the outcomes of stroke attributed to high BMI (5, 9), and these studies provide important information for understanding the health burden of stroke attributed to high BMI. However, few studies have assessed the long-term trend of high BMI-attributed stroke mortality, and the potential effects underlying the temporal trends are still unknown. Furthermore, comprehensive analysis combining past and future trends is still rare. In this study, we used the global burden of disease (GBD) 2019 to analyze the long-term trends of stroke mortality attributed to high BMI in China. To better understand these trends, we also forecasted the future mortality attributed to high BMI. The findings of this study may provide an effective supplement to the existing evidence to help understand the burden of high BMI attributed to stroke in China and provide information to effectively reduce the burden of stroke.

## Methods

### Data sources

Data on stroke attributable to high BMI were obtained from GBD 2019, which was created by GBD collaborators. GBD 2019 provides a systematic assessment of age-, sex-, cause-, and location-specific estimates of 87 risk factor exposures and attributable burdens from 1990 to 2019, covering 204 countries and territories around the world (10). The original data for estimating high BMI in China were collected from a national individual-level survey, where BMI was calculated as weight in kilograms divided by height in meters, and high BMI was defined as BMI ≥ 25 kg/m^2^ for adults (aged 20+ years) (10). Data on stroke mortality were mainly obtained from the Disease Surveillance Points, Maternal and Child Surveillance System, and Chinese Center for Disease Control and Prevention Cause of Death Reporting System (11). Stroke was defined based on the 9th and 10th versions of the International Classification of Disease (ICD10: G45–G46.8, I60–I63.9, I65–I66.9, I67.0–I67.3, I67.5–I67.6, I68.1–I68.2, I69.0–I69.3, ICD9: 430–435.9, 437.0–437.2, 437.5–437.8). GBD 2019 used a comparative risk assessment framework to estimate the stroke mortality attributable to high BMI, which was equal to stroke mortality multiplied by the population attributable fraction (PAF) for high BMI for each age group, sex, and year. The PAF was the proportion of stroke mortality that would be reduced if high BMI was reduced to the level of theoretical minimum risk (BMI, 20–25 kg/m^2^) (10). Age-standardized rates were based on the GBD 2019 global age-standard population (12).

The population data used to estimate the underlying effect of the temporal trends were extracted from GBD 2019. To predict future stroke mortality attributed to high BMI, population data after 2019 were collected from World Population Prospects 2019, which was compiled by the United Nations population division (13).

### Statistical analyses

We used an age-period-cohort (APC) framework to assess the long-term trends of high BMI-attributable stroke mortality and to evaluate the underlying effects of age, period and cohort on these trends. By using the APC framework, the following functions were estimated (14): (1) Drifts, including net drift and local drift, indicates the overall and age-specific annual percentage change of the high BMI-attributable stroke mortality rates over time (11). (2) The longitudinal age curve indicates the expected age-specific high BMI-attributable stroke mortality rate in a reference cohort adjusted for period effects. (3) The cohort (or period) rate ratios (RRs), which represent age-specific rates in each cohort (or period) relative to the reference cohort (or period) (11). Since an individual's birth cohort is calculated by the time period of death and the individual's death age (i.e., birth cohort = period-age), the intrinsic estimator method was used to conduct the APC analyses to solve the problem of identifying model parameters (perfect collinearity of the age, period, and cohort variables) (15).

To conduct APC analysis, the death and population data were arranged into consecutive 5-year periods from 1990–1994 to 2015–2019 (the 2000–2004 survey period was the reference group) and successive 5-year age intervals from 20–24 years to 75–79 years. By following the equation of birth cohort = time period – age groups, we determine 17 consecutive birth cohorts and included those born from 1915–1919 to 1995–1999, with the birth cohort of 1955–1959 used as the reference group. Parameters were estimated by the APC web tool (14) (Biostatistics Branch, National Cancer Institute, Bethesda, MD). The Wald chi-square test was adopted for the significance test of the estimable parameters and functions. All statistical tests were two-sided and performed at the 5% level of statistical significance.

We used the Bayesian APC method to project future mortality and the number of deaths of high BMI-attributable stroke from 2020 to 2030, which have higher coverage (16). The Bayesian APC framework was developed by using the “BAPC” package, which is based on an integrated nested Laplace approximation approach to full Bayesian inference (17). In our study, we used a random walk of second order to model age, period and cohort effects in the Bayesian APC framework. R version 4.1.2 was used to perform statistical analyses.

## Results

### Trends in high BMI-attributable stroke mortality rates by sex from 1990 to 2019

**Figure 1:**
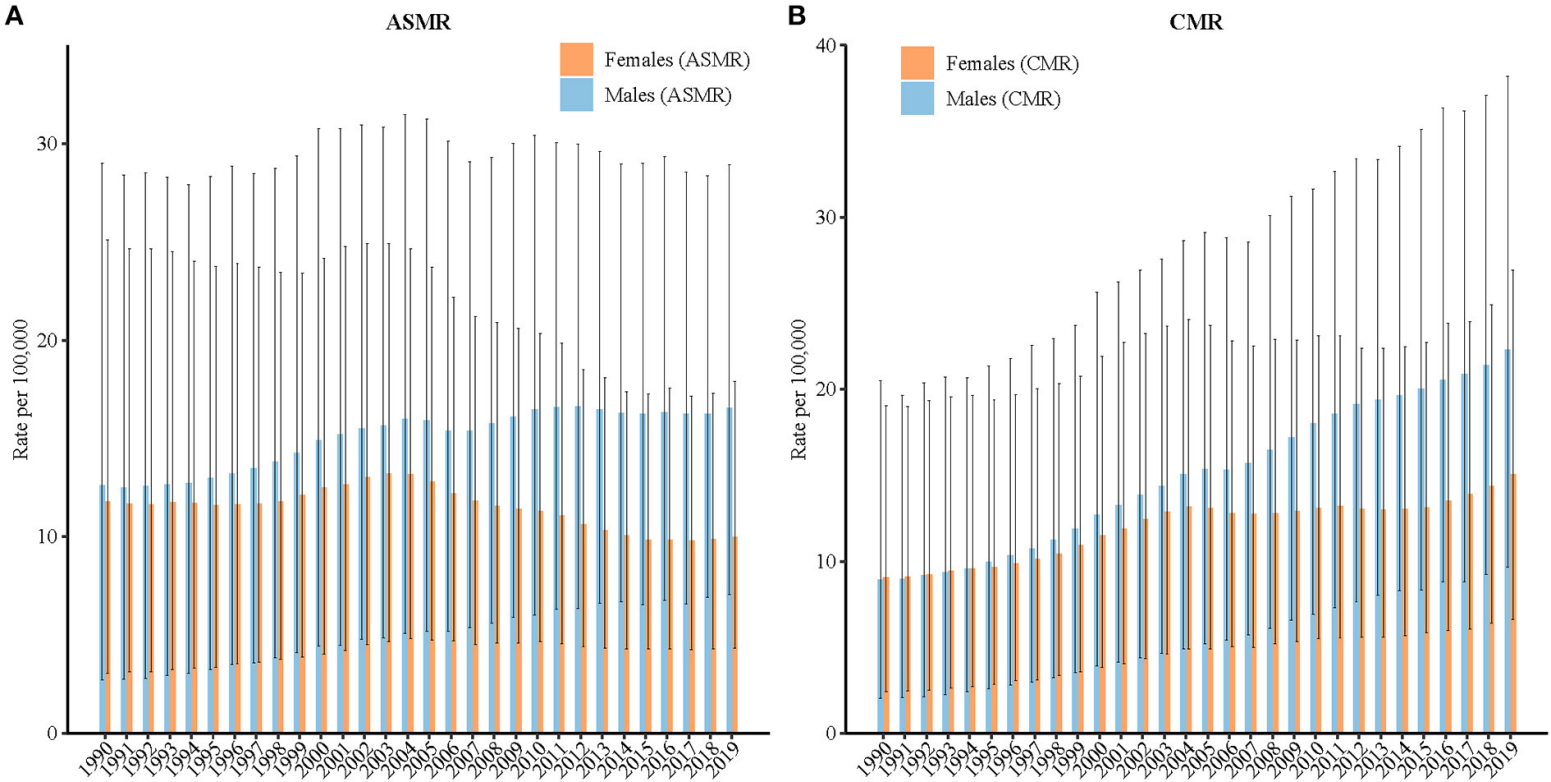
Trends of the **(A)** age-standardized mortality rates (ASMRs) and **(B)** crude mortality rates (CMRs) per 100,000 population for high-BMI-attributable stroke mortality by sex in China, 1990–2019. The ASMR was standardized to the GBD 2019 (Global Burden of Disease Study 2019) global age-standard population.

Figure 1 displays the trends in the crude mortality rate (CMR) and ASMR for high BMI-attributable stroke by sex from 1990 to 2019. The changes in the mortality rates of Chinese males and females suggest that the CMRs in both sexes showed increasing trends, from 8.95 to 22.30 (increased by 149.2%) and from 9.08 to 15.07 (increased by 66.0%) per 100,000 for males and females, respectively. The ASMRs in females decreased from 11.8 to 10.00 per 100,000 (decreased by 15.2%), while in males, the ASMRs increased from 12.6 to 16.5 per 100,000 (increased by 31.1%). Notably, the ASMRs in females increased slightly from 1995 to 2003, reaching its peak in 2003 (13.2 per 100,000 person-years).

### Net drift and local drift values for high BMI-attributable stroke mortality rates in China

**Figure 2:**
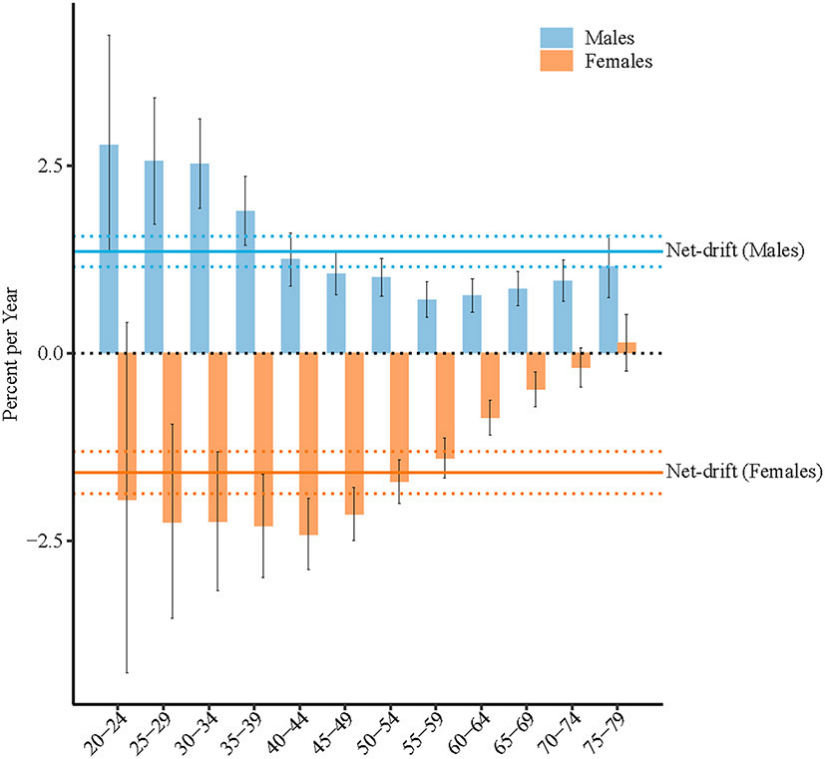
Net drift and local drift values for high-BMI-attributable stroke mortality in China. Age group-specific annual percent change (local drift) with the overall annual percent change (net drift) in high BMI-attributable stroke mortality rate. Net drift values are depicted as solid lines, with dashed lines representing their 95% CIs. Error bars represent the 95% CIs for the local drift values.

Figure 2 displays the net drift (overall annual percentage change) and local drifts (annual percentage changes for each age group) for high BMI-attributable stroke mortality rates in China. The net drifts for high BMI-attributable stroke mortality were 1.4% [95% confidence interval (CI): 1.2–1.6%] and −1.6% (95% CI: −1.9 to −1.3%) for males and females, respectively. The local drift values for high BMI-attributable stroke mortality were above 0 in all age groups for males (*P*<*0.05*) and highest in the 20–24-year group at 2.8% (95% CI, 1.3–4.2%). The local drift values for females with high BMI-attributable stroke mortality were below 0 in the age groups between 25 and 69 years old (*P*<*0.05*) and lowest in the 40–44-year-old group at −2.4% (95% CI: −2.9 to −1.9%).

### Longitudinal age curves of high BMI-attributable stroke mortality by sex in China

**Figure 3:**
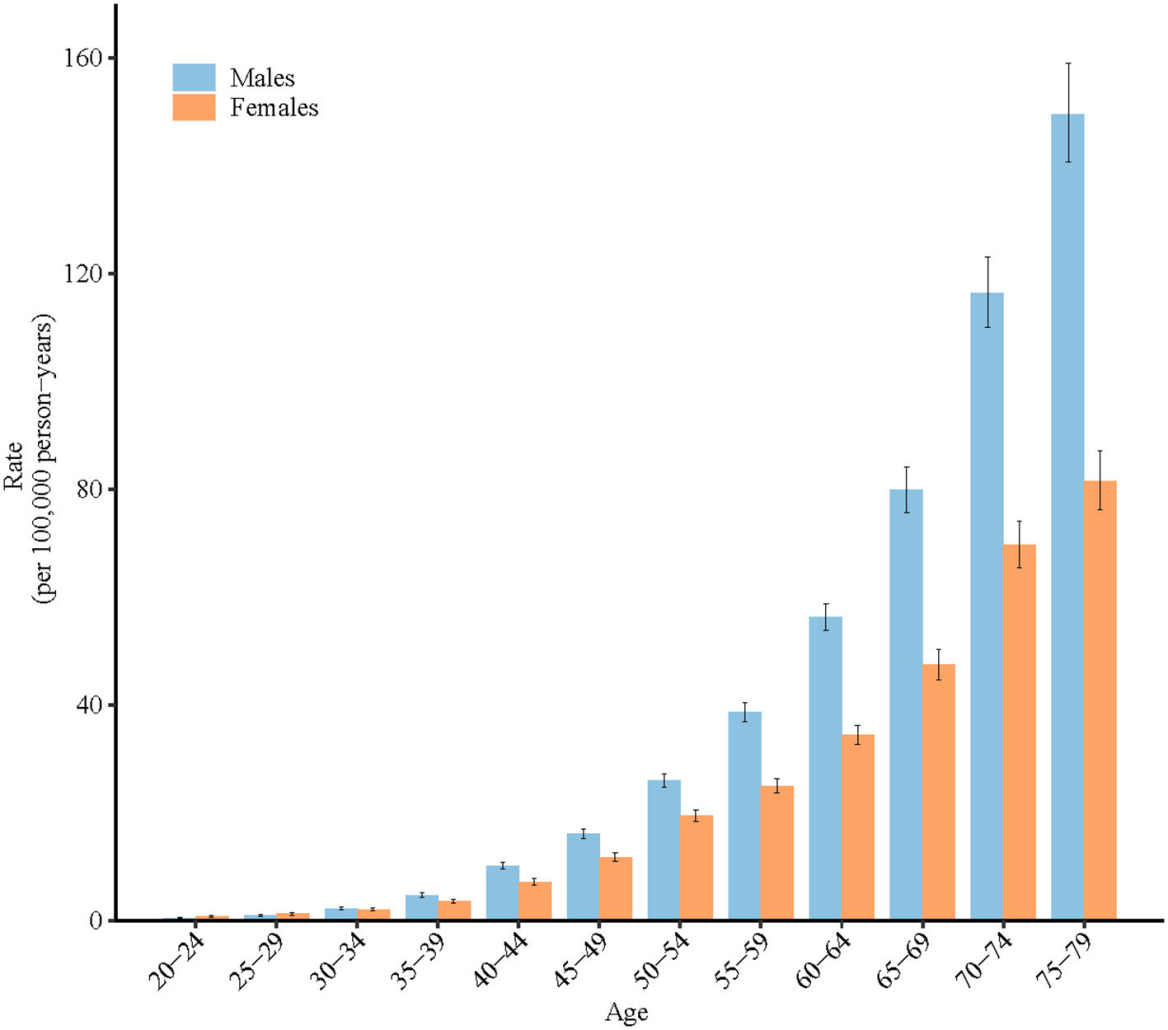
Longitudinal age curves of high-BMI-attributable stroke mortality in China. Fitted longitudinal age-specific rates of obesity-attributable stroke mortality (per 100,000 person-years). Error bars represent the 95% CIs for the longitudinal age curve values.

The longitudinal age curves of high BMI-attributable stroke mortality by sex are displayed in Figure 3. In the same birth cohort, high BMI-attributable stroke mortality rates per 100,000 people increased exponentially to peak at the ages of 75–79 years old for both sexes; rates climbed from 0.5 in men aged 20–24 years to 149.6 in the age group of 75–79 years and from 0.8 to 81.5 for women in the respective age groups.

### Period and cohort RRs of high BMI-attributable stroke mortality rate by sex in China

**Figure 4:**
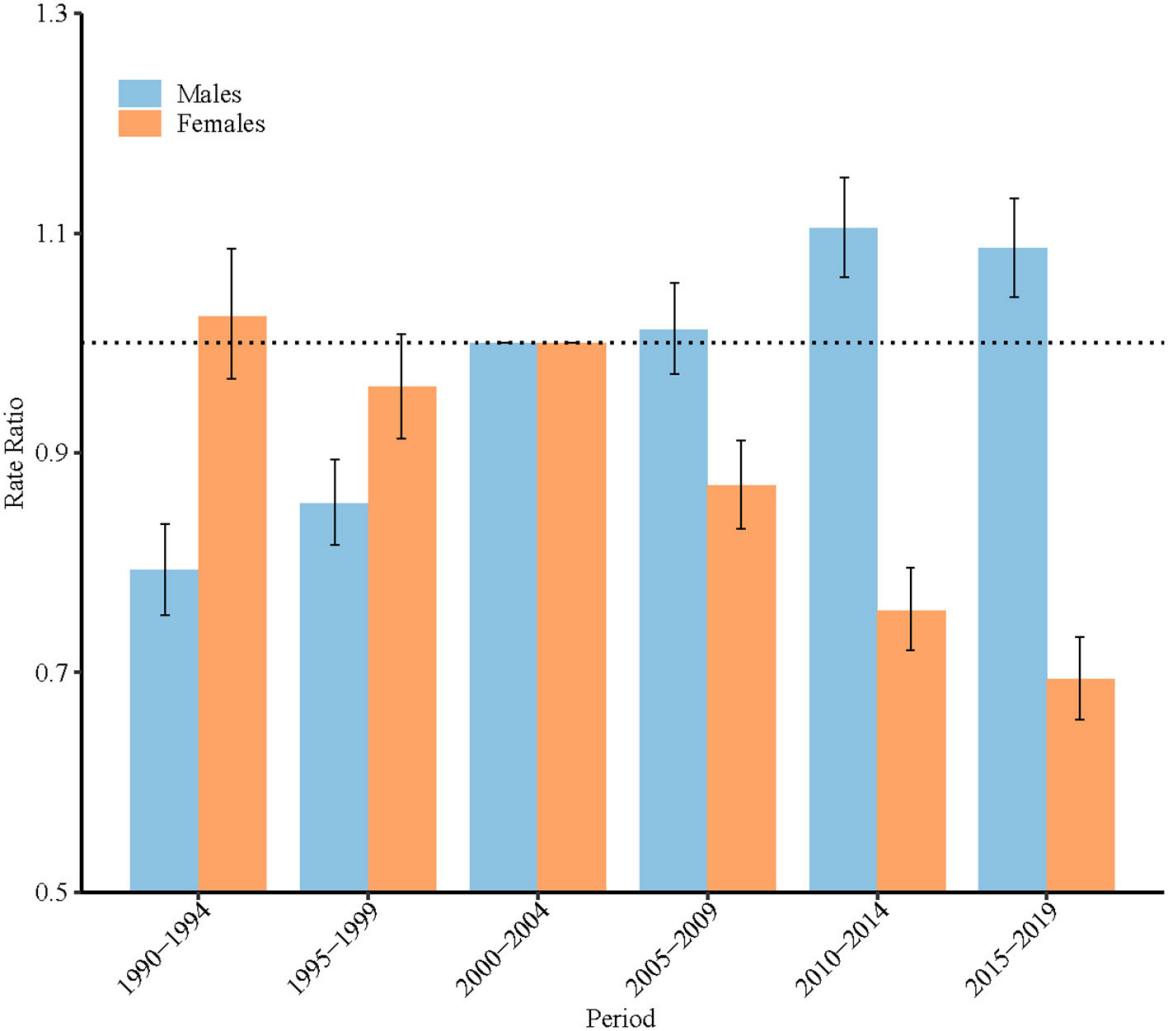
Period relative risks (RRs) of high-BMI-attributable stroke mortality rate by sex in China. The RR of each period compared with the reference period (years 2000–2004) adjusted for age and non-linear cohort effects. Error bars represent the 95% CIs for the RR period.

**Figure 5:**
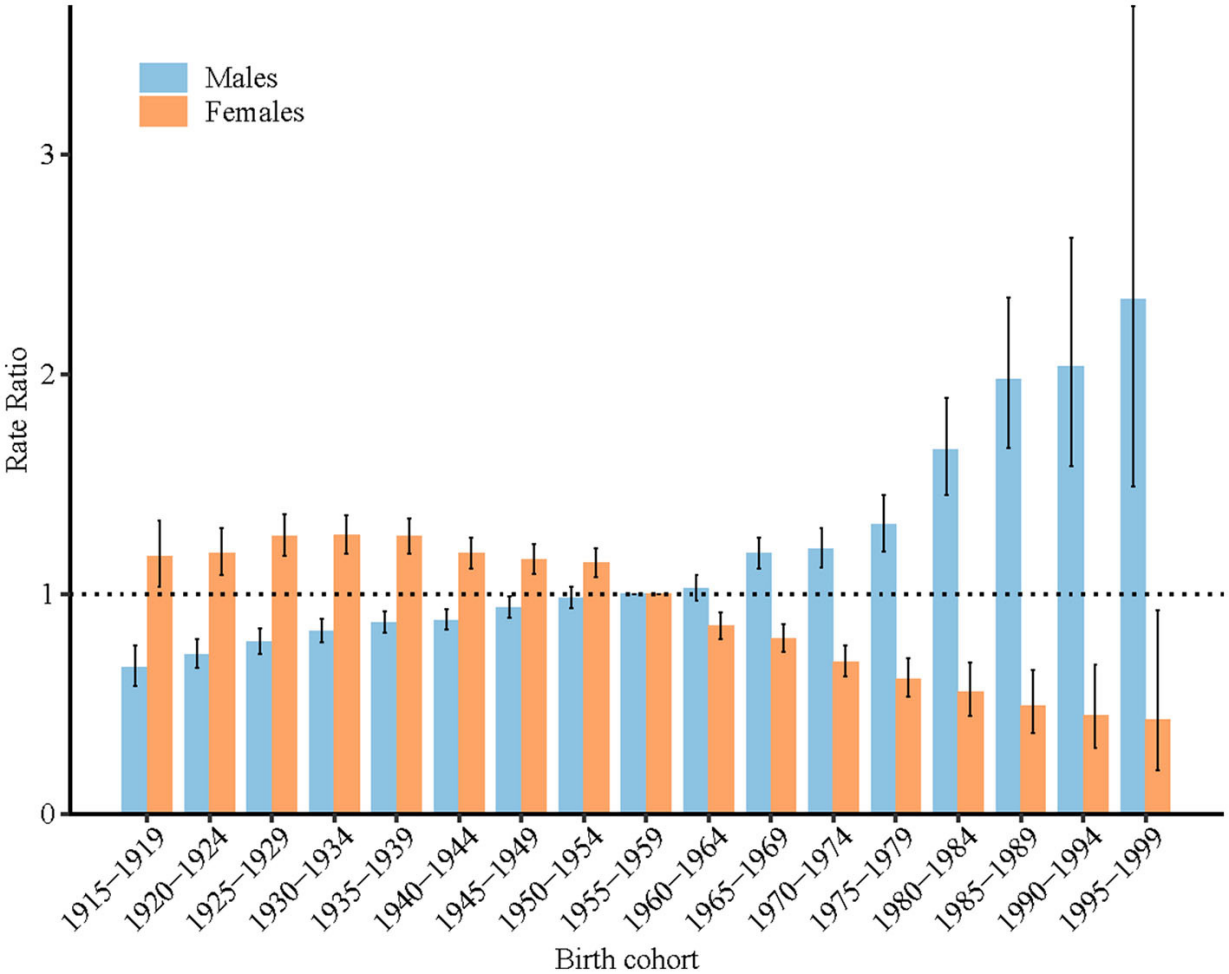
Cohort relative risks (RRs) of high-BMI-attributable stroke mortality rate by sex in China. The RR of each cohort compared with the reference cohort (birth cohort 1953–1957) adjusted for age and non-linear period effects. Error bars represent the 95% CIs for the cohort RR.

According to the results of the Wald test, the cohort and period RRs were statistically significant for both sexes (*P* < 0.001 for all) (Table 1). Figures 4, 5 display the estimated period and cohort RRs for high BMI-attributable stroke mortality rates by sex. The period RRs after the reference period showed a downward trend in females. In contrast, the period RRs of men showed an upward trend, but this increase was halted in the most recent period (Figure 4). Similarly, in all consecutive cohorts, the cohort RRs showed a monotonic increasing pattern in males, while the female cohort RRs showed a downward trend after the birth cohort 1930–1934 (Figure 5).

**Table 1:** The Wald chi-square test for estimable functions in the APC model.

**Null hypothesis**	**Male**		**Female**	
	**Chi-square**	***P*** **-value**	**Chi-square**	***P*** **-value**
All period RR = 1	191.509	< 0.001	195.219	< 0.001
All cohort RR = 1	230.405	< 0.001	232.983	< 0.001
All local drifts = Net drift	43.770	< 0.001	108.496	< 0.001

RR, Rate ratio; Net drift, annual percentage change in the expected age-adjusted rates over time; Local drift, annual percentage change in the expected age-specific rates over time.

### Projection of high BMI-attributable stroke mortality

**Figure 6:**
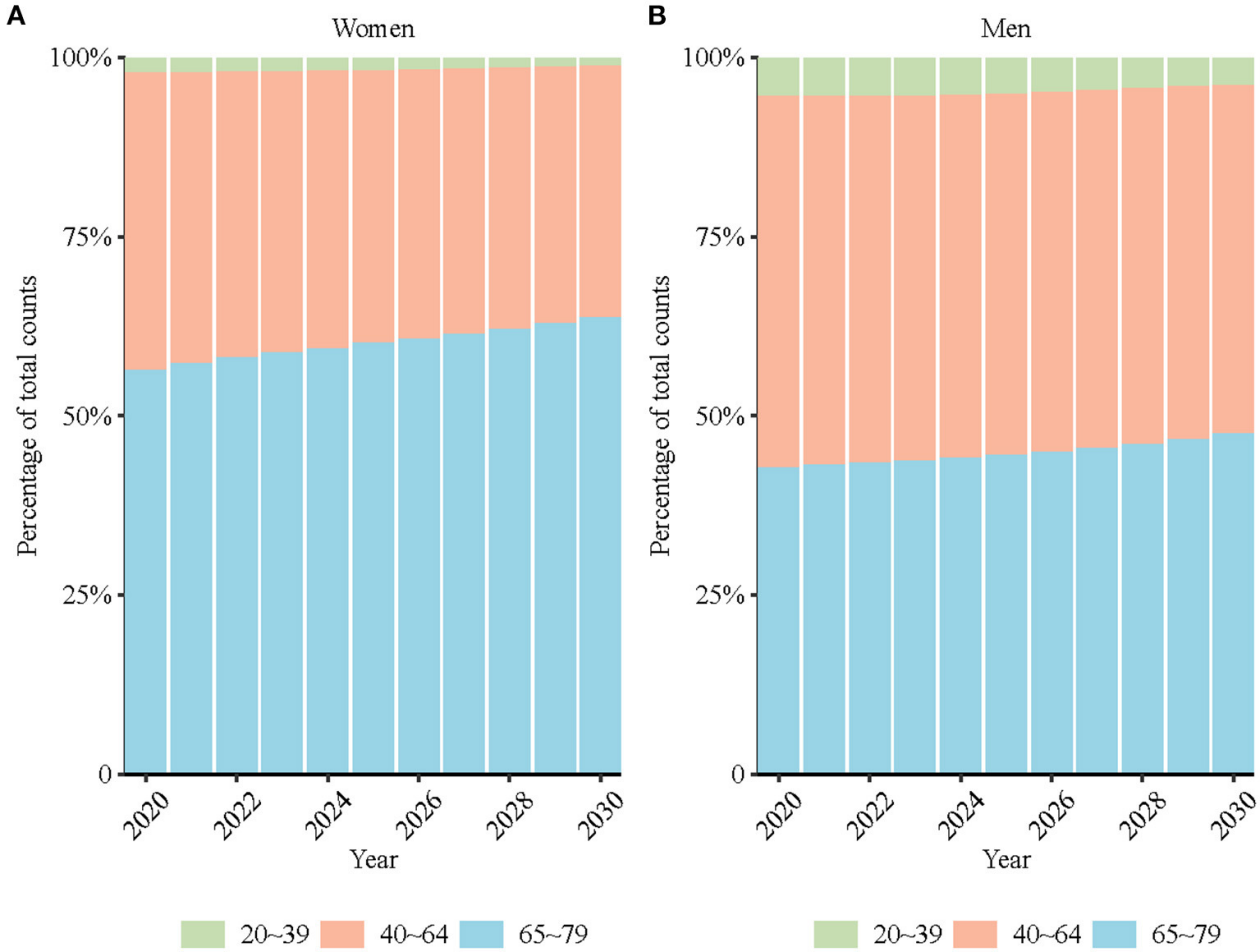
Percentage of projected obesity-attributable stroke deaths for women **(A)** and men **(B)** in three age groups (20–39, 40–64, 65–79).

Table 2 shows the projected ASMR of high BMI-attributable stroke in participants aged 20–79 years. The high BMI-attributable stroke mortality was projected to increase from 25.6 (95% CI: 25.5, 25.8) per 100,000 people in 2019 to 26.9 (95% CI: 14.0, 39.8) in 2030 in males (increased by 5.1%) and from 15.3 (95% CI: 15.2, 15.4) per 100,000 people in 2019 to 13.9 (95% CI: 6.4, 21.4) in 2030 in females (decreased by 9.2%). The proportion of elderly individuals with high BMI-attributable stroke mortality was projected to increase in both males and females (Figure 6).

**Table 2:** Projected obesity-attributable stroke mortality rate by sex (20–79 years).

	**Mortality rate in 2019^a^ (95%CI)**	**Mortality rate in 2030^a^ (95%CI)**	**Change (%)^b^**
Female	15.3 (15.2, 15.4)	13.9 (6.4, 21.4)	−9.2
Male	25.6 (25.5, 25.8)	26.9 (14.0 39.8)	+5.1

a The mortality rate was weighted by the GBD 2019 age-standardized population.

b The change value in mortality from 2019 to 2030 divided by the mortality value in 2019.

## Discussion

In the past 30 years, the ASMR for high BMI-attributable stroke decreased by 15.2% in females and increased by 31.1% in males. The mortality rates increased for each age group from 20 to 79 years old in males and decreased for each age group from 25 to 69 years old in females. The risk of death due to high BMI-attributable stroke in females born after the 1930–1934 birth cohort continued to decrease as the birth year moved forward, but it continued to increase in males. The ASMR for high BMI-attributable stroke in 2030 was projected to decrease in females but rise in males. The proportion of elderly individuals with high BMI-attributable stroke mortality was projected to increase in both males and females.

The impact of high BMI on stroke outcomes is mainly concentrated in the cerebrovascular system and blood–brain barrier (18), and this may be related to the multiple mechanisms. First, inflammation plays an important role in mediating obesity-related stroke risk. Overweight and obesity can lead to “low-grade chronic inflammation”, which increases the concentrations of C-reactive protein (CRP) and interleukin (IL)-6 in plasma (18, 19). High CRP and IL-6 concentrations are risk factors for stroke. Although the concentrations of proinflammatory markers are relatively low by comparison, they are maintained over long periods. Second, overweight and obesity also aggravate inflammatory damage to cerebral microvessels. This leads to vascular wall remodeling and brain blood flow changes and triggers a prethrombotic state (18). In addition, overweight and obesity can induce the early onset and accumulation of cerebrovascular risk factors that are closely related to metabolic disorders, such as insulin resistance, diabetes, hypertension and dyslipidemia. Overweight and obesity, together with these risk factors, can lead to the progression of atherosclerosis or thromboembolism. These conditions may result in arterial occlusion or rupture, thus leading to stroke (5, 19). Obesity may also remodel the middle cerebral arteries. The diameter of the lumen decreases, the thickness of the vascular wall increases, and vascular tension changes result in an increase in vascular resistance. This increase may lead to cerebrovascular dysfunction. The autoregulation of cerebral blood vessels is also impaired in obese patients (18). Increased oxidative stress and increased insulin resistance due to high BMI also accelerate atherosclerosis, which increases the risk of stroke (20). Moreover, a lack of physical activity has also been identified as a risk factor for stroke in China (1). This may have a synergistic effect with obesity in inducing stroke.

In the past 30 years, the CMR for high BMI-attributable stroke in China showed increasing trends in both sexes, which is consistent with the trend of the CMR for stroke in China (11); however, a difference was found in the ASMR of stroke in men. Unlike the overall decreasing trends of ASMR of stroke, that of high BMI-induced stroke showed an overall upward trend in men. This may be related to the continuous and rapid increase in the prevalence of overweight and obesity among Chinese men. Previous studies showed that from 1991 to 2015, the prevalence of overweight and obesity in Chinese men increased by 282.1 and 875.0%, respectively, much higher than the 108.4 and 282.4% increase in women (8). In addition, evidence has shown that for younger people (under 65 years old), the risk of stroke among overweight men is higher than that among overweight women [hazard ratio (HR): 1.98 vs. 1.38] compared with normal weight individuals (21). In addition, hemorrhagic stroke, the main subtype of stroke that causes death (22), has a much higher HR in overweight men than in overweight women (HR: 3.62 vs. 2.06) (21). Moreover, the risk of stroke among males was always higher than that among females (HR: 1.0 vs. 0.78) (23).

Consistent with the age effect of stroke mortality (22), the risk of death from stroke due to high BMI increases exponentially with age, especially among people over 65 years old. Age is the most important demographic risk factor for stroke. The stroke mortality of people over 65 years old in China accounts for more than 70% of the stroke mortality of the whole population (15). Considering the large aging population of China (22) and the increase in the size of the elderly obesity population (8), high BMI-attributable stroke may place an enormous burden on health systems throughout China.

The period effect demonstrated the direct impact of external factors that equally affect all age groups at a particular calendar time, and the cohort effect reflects different disease risks in various birth cohorts. In this study, the period effect of high BMI-attributable stroke mortality in women decreased after 2000–2004, and the cohort effect decreased after the 1930–1934 birth cohort. The decreasing trends of period and cohort effects may be related to improvements in medical care and techniques (11). In addition, with the process of urbanization and the development of universal health care, the accessibility and affordability of medical services have improved (22). The proportion of the population living in urban areas increased from 26% in 1990 to 50% in 2010, and the coverage of basic medical services reached more than 90% in 2013, which effectively reduced the risk of death from stroke in China (22, 24). Although the prevalence of obesity in China has continued to increase over the past 30 years, the increase in the prevalence of obesity among females is small (4, 8), and the unfavorable effect from these increases seems to not be strong enough to reverse the decreasing trends of stroke mortality. It is expected that the high BMI-attributable stroke mortality in women will continue to decrease in the future 10 years.

Unlike the decreasing trends of period and cohort effect of stroke mortality in females (11), the cohort effect of high BMI-attributable stroke mortality continually increased in males. This may mainly relate to the continually increasing burden of obesity among males in China. As described above, men in China have exhibited a significant increase in high BMI prevalence over the last two decades. According to the World Health Organization recommended BMI classifications (overweight defined as BMI ≥ 25), nearly half of Chinese males were overweight in 2015 (22). In addition, some stroke risk factors (e.g., smoking) have a synergistic effect on the relationship between body mass index and stroke (25). The proportion of smokers among Chinese males was high, consistently reaching nearly half of Chinese males (47.2% in 2013) (26). Notably, according to the current trend, the mortality related to high BMI among Chinese males is expected to continue to increase. To complicate matters further, the proportion of elderly patients with high BMI-attributable stroke mortality was projected to increase in both males and females, which means that more effective interventions are urgently needed to prepare for the future stroke burden on elderly individuals.

The most recent national survey shows that the overweight and obesity rate has exceeded 50% in China (7). According to China's overweight and obesity standard, China may have surpassed the United States and become the country with the largest number of individuals with obesity in the world (27). However, it is worrisome that many people with high BMI in China do not have an accurate weight perception (28). More than 50% of adults with high BMI believe that they are underweight or normal weight (27, 29). Moreover, even among the people with high BMI who have a correct understanding of high BMI, more than 60% of them did not take action to lose weight. This phenomenon is particularly true among males, elderly individuals, and less-educated people (28). Therefore, implementing a comprehensive health education plan is of great importance to cultivate accurate and healthy body management awareness among people.

This study was subject to some limitations. First, as this study is based on statistical data from 1990 to 2019, it is not a cohort study. As with other studies using the same analysis method, the interpretation of the research results at the population level may not be applicable at the individual level. Therefore, further study at the individual level is needed to confirm the research results. Second, due to the limitation of data resources, we did not explore the stroke mortality attributed to high BMI in urban and rural areas in China. Considering the rapid growth of the prevalence of overweight and obesity in rural areas of China and the relatively weak medical capacity (27, 30), it is necessary to evaluate high BMI-attributed stroke mortality in urban and rural areas in China.

## Conclusions

Over the past three decades, the ASMR of stroke attributed to high BMI has decreased among females in China, and this decreasing tendency is projected to continue in the future. Unlike that among women, the ASMR of stroke attributed to high BMI among Chinese males has increased over the past 30 years, and it is projected to continue to increase in the future. The proportion of older people who die from high BMI-attributed stroke was projected to gradually increase in both sexes. Considering that there was no evidence that the aging process will slow in China, high BMI-attributed stroke may have a strong impact on the health of the elderly Chinese population. More effective efforts are needed, especially for males and elderly individuals.

## Data availability statement

The original contributions presented in the study are included in the article/supplementary material, further inquiries can be directed to the corresponding authors.

## Ethics statement

The GBD study uses de-identified, aggregated data. Therefore, a waiver of informed consent was reviewed and approved by the University of Washington Institutional Review Board.

## Author contributions

MC designed the research and performed the data-analysis. MC, ZD, WZ, YZ, SL, and HC drafted the original manuscript. ZD and ZS critically revised the manuscript. ZS and MC provided administrative support for the project and had primary responsibility for the final manuscript. All authors read and approved the final manuscript.

## Funding

This work was financially supported by Shaanxi Provincial National Science Foundation (2022JQ-905), Shaanxi Provincial People's Hospital Talent Funding Project (2021JY-12), and Xinghuo Talent Program of Nanjing First Hospital.

## Conflict of interest

The authors declare that the research was conducted in the absence of any commercial or financial relationships that could be construed as a potential conflict of interest.

## Publisher's note

All claims expressed in this article are solely those of the authors and do not necessarily represent those of their affiliated organizations, or those of the publisher, the editors and the reviewers. Any product that may be evaluated in this article, or claim that may be made by its manufacturer, is not guaranteed or endorsed by the publisher.
